# Addressing inequity to achieve the maternal and child health millennium development goals: looking beyond averages

**DOI:** 10.1186/1471-2458-12-1119

**Published:** 2012-12-27

**Authors:** George M Ruhago, Frida N Ngalesoni, Ole F Norheim

**Affiliations:** 1 School of Public Health and Social Sciences, Muhimbili University, P.O Box 65015, Dar es Salaam, Tanzania; 2 Ministry of Health and Social Welfare, P.O Box 9083, Dar es Salaam, Tanzania; 3 Department of Public Health and Primary Health Care and Centre for International Health Kalfarveien 18, University of Bergen, Bergen, 5018, Norway

## Abstract

**Background:**

Inequity in access to and use of child and maternal health interventions is impeding progress towards the maternal and child health Millennium Development Goals. This study explores the potential health gains and equity impact if a set of priority interventions for mothers and under fives were scaled up to reach national universal coverage targets for MDGs in Tanzania.

**Methods:**

We used the Lives Saved Tool (LiST) to estimate potential reductions in maternal and child mortality and the number of lives saved across wealth quintiles and between rural and urban settings. High impact maternal and child health interventions were modelled for a five-year scale up, by linking intervention coverage, effectiveness and cause of mortality using data from Tanzania. Concentration curves were drawn and the concentration index estimated to measure the equity impact of the scale up.

**Results:**

In the poorest population quintiles in Tanzania, the lives of more than twice as many mothers and under-fives were likely to be saved, compared to the richest quintile. Scaling up coverage to equal levels across quintiles would reduce inequality in maternal and child mortality from a pro rich concentration index of −0.11 (maternal) and −0.12 (children) to a more equitable concentration index of −0,03 and −0.03 respectively. In rural areas, there would likely be an eight times greater reduction in maternal deaths than in urban areas and a five times greater reduction in child deaths than in urban areas.

**Conclusions:**

Scaling up priority maternal and child health interventions to equal levels would potentially save far more lives in the poorest populations, and would accelerate equitable progress towards maternal and child health MDGs.

## Background

In September 2000, global leaders gathered at the United Nations assembly and adopted a resolution on the Millennium Development Goals (MDG). Among the main objectives is a two-thirds reduction in child mortality in the under-fives (MDG 4) and a three-quarter reduction in maternal mortality (MDG 5) relative to 1990 rates [1]. Progress towards MDG 4 and 5 is promising with significant acceleration globally [2,3]. However, some developing countries are still lagging behind. In Tanzania, there have been substantial reductions in maternal and child mortality. Under-fives mortality declined from 141 deaths per 1000 live births in 1990 to 81 in 2010, maternal mortality has dropped from 578 deaths per 100,000 live births in 1990 to 452 in 2010 [3,4]. But these reductions are well short of Tanzania’s MDG targets of 54 deaths per 1000 live births and 193 deaths per 100,000 live births for MGD 4 and 5 respectively.

Inequity in access to and use of child and maternal health interventions has been highlighted as hindering progress towards child and maternal health MDGs [5]. A 2010 UNICEF report on progress for children showed that in half the developing countries which had an overall reduction in under-five mortality, inequality in under-five mortality between the poorest and the richest households increased by more than 10 per cent [6]. However the disparity in mortality is masked by national average data. In the least developed countries accounting for more than 90 percent of maternal and child mortality globally, there is inequity in coverage of key health interventions, with a country mean coverage gap of 43 among the poorest and wealthiest quintiles of the population [7]. In Tanzania, there is, on average, a 60 percent coverage gap in access to health facilities and skilled birth attendants. The richest populations enjoy 90 percent coverage compared with only 33 percent for the poorest population [8]. Numerous studies have showed that health systems are consistently unjust: likely to provide more and higher quality services to the well-off compared to the poor [9,10]. Health inequities are a consequence of high levels of direct and indirect payment for services, unfair distribution of economic resources, and unequal political and social authority between groups in society [11]. Analysis of equity trends in health outcomes can guide effective and fair service delivery strategies [12]. Therefore it is important to generate evidence about inequity that can inform decision making and priority setting.

Many countries in Sub-Saharan Africa, such as Tanzania, make limited use of scientific evidence to inform policy debate and health care priority setting. Inadequate use of the evidence contributes to inequity in access to and use of child and maternal health interventions and health outcomes. In order to reach MGDs targets, scale up of health interventions is essential. To achieve rapid scale up requires evidence on what works and with what resources. This can guide policy makers and governments in identifying, prioritizing and implementing high impact health interventions [13]. However, targets for the Millennium Development Goals for maternal and child health interventions are set on the basis of national average data. In a recent work, Reidpath et al. [14], used a hypothetical country to show that the use of national average data can conceal inequities in mortality between social and economic groups. Expanding intervention coverage using national average data may not address existing disparities in coverage between socioeconomic groups or geographical locations [15]. In order for the health system to achieve universal coverage, it is important that any scale up addresses the needs of all population groups across geographical locations and socioeconomic status by disaggregating coverage data to reflect distinct groups within society.

Tools such as the Lives Saved Tool (LiST) are useful to policymakers in priority setting. The tool can be used to identify which interventions can be scaled up rapidly and what their impact on mortality may be [16,17]. LiST can also be used to address health distributional impact across household wealth quintiles [18]. Rational, equitable and evidence based priority setting is key to increasing the coverage of accessible and essential health care interventions. The aim of this paper is to estimate the potential health gains and equity impact if coverage of a set of high impact priority interventions for mothers and under fives were scaled up to the national universal coverage targets for achieving MDGs in Tanzania.

## Methods

### Data sources

We use disaggregated data from Tanzania to reflect mortality and coverage in five wealth quintiles from the poorest to the richest and in rural and urban areas. Baseline coverage and mortality data for this study were extracted from the openly available, 2010 Tanzania Demographic and Health Survey (TDHS) [8]. Permission to conduct research was sought and obtained from the Tanzania National Institute of Medical Research (NIMR). We define universal coverage as 80–90% coverage, acknowledging that the ideal 100% coverage may be hard to reach. For endpoint coverage, we used targets from the 2008 Tanzania National Strategic Plan for reduction of maternal, newborn and child mortality (90% for most targets) [19]. In case national targets were lower than the current TDHS 2010 coverage levels in any of the sub-national or socioeconomic groups, TDHS data were used as endpoint coverage. Table 1 below provides a summary of interventions, coverage estimates and targets.

**Table 1:** Intervention coverage (%) for maternal and child health interventions by wealth quintiles and geographical residence used as input in LiST

				**Wealth quintiles**					
**Interventions**	**National**	**Urban**	**Rural**	**Poorest**	**Poor**	**Middle**	**Less Poor**	**Richest**	**Targets**
**Pregnancy and child birth care**									
Antenatal Care	43.0	54.8	39.1	39.1	39.1	43.0	54.8	54.8	90
Facility based delivery	50.5	83.0	42.0	33.1	36.2	45.8	62.5	89.6	90
Skilled birth attendance	50.5	83.3	42.3	33.0	35.8	47.0	63.3	90.4	90
**Diarrhoea management**									
Oral Rehydration Salt (ORS)	44.2	44.4	44.0	40.8	42.6	43.3	54.0	38.3	90
**Pneumonia management**									
Case Management of Pneumonia	42.6	45.6	36.6	34.7	37.0	36.7	39.7	48.7	80
**Malaria**									
Insecticide Treated Nets (ITN)	63.4	64.9	63.0	56.6	63.9	63.6	66.8	68.0	80
artemisinin-based combination therapy	37.6	33.2	39.1	44.1	36.0	35.6	32.1	36.0	80
**Population (%) to national population**	100	26	74	19	21.5	21.9	19.7	17.5	

### Data analysis

We used the Lives Saved Tool (LiST) version 4.47 for modeling. LiST is free, downloadable software and is part of the spectrum policy modeling system developed by the John Hopkins University [20]. The tool was used to model the potential health impact of scaling up priority health interventions on maternal and child mortality for a period of five years. In this study, the baseline year is 2011 and the final year is set at the target for Millennium Development Goals, 2015.

LiST is pre-loaded with country specific average data. To allow for wealth quintile and urban vs. rural analysis, we adjusted the national demographic projection to obtain population estimates for each of the five wealth quintiles as well as urban and rural areas. In other words, we partitioned the whole population into seven “sub-populations” or sub-groups. The national total fertility rate was adjusted by the five wealth quintiles and urban/rural estimates of fertility rates from Tanzanian health and demographic surveys from 1992 to 2010. The adjusted fertility rate was applied from the first year of population to the target year. The proportion of each of the quintiles, urban/rural areas to the total national population was multiplied by the first year population of the national population estimates preloaded in LiST to estimate each of the sub-group populations. Migration values were adjusted to zero. The maternal mortality ratio and under-fives mortality rates by SES quintile and urban/rural were updated for the sub-group analysis using current data from TDHS 2010. Default data for cause-specific mortality was used. However, we assumed that the higher/lower than average neonatal, infant and under-five mortality rates in each quintile reported in demographic and health survey were distributed in proportion to the original distribution of cause-specific mortality. The family planning module was updated, the total fertility rate and the unmet need for family planning was adjusted to reflect the sub-group current data. The LiST user manual provides detailed procedures for sub-group modeling [21]. The data on the effectiveness of interventions are default in LiST, updated frequently from comprehensive reviews under the Child Health Epidemiology Reference Group (CHERG) [22].

We entered the baseline coverage for each quintile, urban/rural and national level for a set of high impact priority interventions for maternal health (skilled birth attendance and health facility delivery, as proxy predictors of Basic Emergency Obstetric Care and Comprehensive Emergency Obstetric Care) into LiST. Similarly, coverage data per quintile and urban/rural for child health interventions (oral rehydration salts (ORS) for diarrhoea management, antibiotic for pneumonia treatment, Insecticide Treated Nets (ITN) and artemisinin-based combination therapy (ACTs) for the management of malaria) were entered.

The TDHS 2010, does not report maternal mortality by wealth quintile, so the lowest, midpoint and high estimates were used for quintiles. To account for any possible biases the two lowest quintiles (40%) likely to have higher maternal mortality were assigned with the highest estimates of maternal mortality ratio. The modeling exercises were done by linking intervention coverage, effectiveness and cause of mortality. We observed the expected change of mortality in maternal and under-fives and lives saved over the five-year period. Details on the assumptions built into the LiST module have been well documented elsewhere [23,24].

### Equity analysis

**Figure 1:**
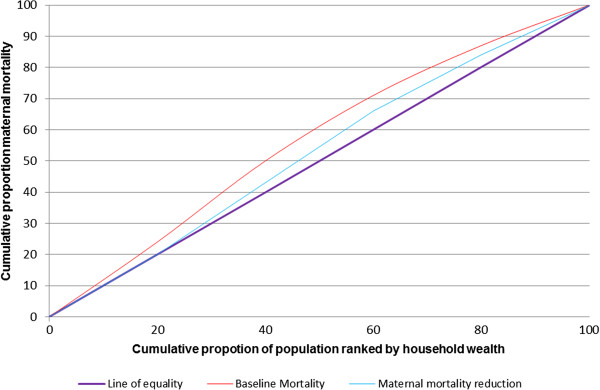
Degree of inequality in maternal mortality.

**Figure 2:**
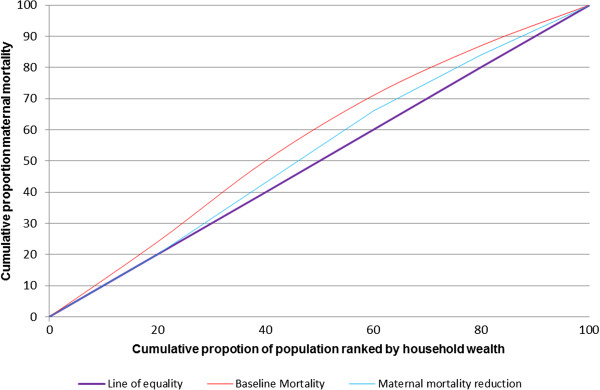
Degree of inequality in under five children mortality.

Concentration curve and concentration index were used to measure the equity impact of the priority intervention scale up. A concentration curve is used to display the distributional impact of wealth related inequity in MMR and U5M, (Figures 1 and 2). The baseline and endpoint mortality measured before and after intervention scale up (maternal or under five mortality) were cumulatively plotted on the *y*-axis, against the cumulative proportion of (mothers or under-fives) population ranked by their socioeconomic status from lowest to highest on the x axis. When the curve lies on the line of equality, all mothers or under fives, regardless of their socioeconomic status have the same mortality. If it lies above the line of equality, mortality is more prominent amongst the poorest population, indicating a pro-rich distribution. On the other hand if the curve lies below the line of equality, this indicates lower mortality in the poorest population, hence a pro poor distribution. To obtain the magnitude of inequality, we used the concentration index [25]. The measure ranges from −1 to 1, with a zero index indicating no wealth related inequity and a negative index indicating higher maternal or under five mortality among the poor.

## Results

Table 2 below shows changes in the maternal mortality ratio and deaths averted as a result of the scale up of high impact priority interventions for maternal health.

**Table 2:** Maternal mortality ratio for five quintiles, at baseline (No coverage change), and modeled for endpoint (Coverage change with priority Interventions) using LiST

	**Mortality reduction (per 100,000 live births)**			**Maternal life saved**		
**Population Level**	**Baseline (No coverage change) MMR 2011**	**Endpoint (Coverage change) MMR 2015**	**Mortality ratio reduction (N)**	**Baseline (2011)**	**Endpoint (2015)**	**Deaths averted by 2015**
Richest	353	197	156	925	517	408
Less Poor	353	193	160	1789	761	1028
Poor	454	224	230	2722	1096	1626
Very Poor	556	271	285	2814	1372	1442
Poorest	556	270	286	2568	1255	1213
**Conc. index**	**−0.105**		**−0.032**			
Urban	353	192	45.6	1289	700	589
Rural	556	273	51.0	9740	4785	4955
National	452	248	204	9787	5146	4641
**MDG Target**	**193**					

The scaling up of interventions by wealth quintile towards equal and universal coverage achieved a significant reduction in maternal mortality: the poorest population benefiting the most, with a reduction in mortality ratio of 286 per 100,000 live births compared with only 156 in the richest quintile. In all, targeting the poorest population saves three times more maternal deaths compared to targeting the richest quintile. That corresponds to a reduction in inequality from a pro rich concentration index of −0.11 to a more equitable concentration index of −0.03. The pro-poor reduction in mortality is depicted by the concentration curve (Figure 1).

Scaling up rural maternal health interventions to the current coverage level accessible to the urban and richest populations (90%) is likely to avert eight times more maternal deaths, i.e., 4955 deaths averted in rural areas compared to 589 in urban areas.

Table 3 above, describes the outcome of scaling up priority interventions for the three leading causes of mortality in under-fives in Tanzania (diarrhoea, pneumonia and malaria). Increasing coverage levels of health interventions in the poorest under-fives to the same coverage level as the richest quintiles (Table 1) in a period of five years is likely to reduce under-five mortality in the poorest children by 43 per 1000 live births, compared with 31 in the richest population. The poorest population is likely to avert more than twice the number of under-five deaths, ie, 18974 in the poorest group compared to 7949 in the richest. The concentration curve (Figure 2), portrays the pro-poor reduction in mortality from baseline concentration index −0.12 to a near perfect equality index at endpoint −0.03.

**Table 3:** Under Five mortality rates for five population levels, at baseline (No coverage change), modeled for endpoint (Coverage change with priority Interventions) using LiST

	**Mortality reduction (per 1000 live births)**			**Under five life saved**		
**Population Level**	**Baseline (No coverage change) U5MR (2011)**	**Endpoint (coverage change) U5MR (2015)**	**Mortality reduction (N)**	**Baseline mortality (2011)**	**Endpoint mortality (2015)**	**Deaths averted by 2015**
Richest	84	53	31	21534	13585	7949
Less Poor	88	55	33	33599	21105	12494
Poor	91	53	38	41346	25268	16078
Very Poor	92	54	38	44941	26413	18528
Poorest	103	60	43	45803	26829	18974
**Conc. Index**	**−0.119**		**−0.027**			
Urban	94	58	36	32984	20640	12344
Rural	92	54	38	153087	91240	61847
National	81	48	33	164818	100726	64092
**MDG Target**	**54**					

The scale up of health interventions for the under-fives in rural and urban areas to the same coverage levels of 80 and 90%, over a period of five years, reduce five times more deaths, i.e., 61847 in rural areas compared to 12344 in urban areas.

## Discussion

The results of this study show that using wealth and rural/urban disaggregated intervention coverage in models can guide policy makers on health outcomes and equity impact of scaling up effective interventions in different population groups. The scale up of health intervention coverage to universal levels of 80 to 90% has potential positive distributional impacts for the worst-off populations and may accelerate equitable achievement of maternal and child Millennium Development Goals. This study has shown that if the wealth and geography-related gap in coverage of a set of high impact priority health interventions is redressed, the under-five mortality rate will be reduced more equitably, may even exceed the target for Millennium Development Goals in Tanzania. Services for the poorest groups would save three times more children compared to the richest groups. The reduction in maternal mortality to the MDG target in Tanzania would be likely to be achieved only by the two richest quintiles, but there would be less inequality in mortality. Rural areas would see a reduction in maternal deaths of eight times that in urban areas, and a reduction in child deaths five times that of urban areas if interventions were scaled-up. At the current coverage, without rapid intervention scale up in Tanzania, MDG 4 is likely to be achieved by 2030 and MDG 5 after 2040 [3]. Therefore, investing in the health of the poorest households and populations in rural areas, and scaling up a few high impact priority interventions could be fundamental to achieving the MDGs. These findings are consistent with those of earlier studies that highlighted the need to address inequity concerns in health care to speed up achievement of the health related MDGs [5,14,26-28].

Addressing inequity is also in line with universal health care policy now being promoted by many UN organizations, public health initiatives, as well as the Tanzanian government [15,29-31]. To succeed in providing universal health coverage, a health system requires qualified human resources, a functioning logistic and supply system, health information systems to assist monitoring and evaluation, good governance and appropriate resource allocation. Shortages of and unequal distribution of human resources for health between urban and rural districts, (the former reported to have more than twice the number of qualified health professionals as the latter), diminishes the chances of reaching the under-served in developing countries such as Tanzania [32,33]. Reinforcing primary care with qualified health workers and strengthening the health system through direct investments in primary health care, with a focus on community health worker in hard to reach areas and in areas with high poverty is important so that universal coverage can reach the poorest populations and reduce inequities in maternal and under-five health outcomes. We believe sub-group analysis in LiST, as demonstrated in this article, is indispensable for making the right decisions at all levels of a health system. Focusing only on average levels of intervention coverage and mortality fails to capture important distributional information which is crucial to strategic decisions for achieving the Millennium Development Goals. A recent study by Carrera, C., et al. has revealed that, health policies addressing geographical and wealth related inequity in child healh intervention are cost effective and reduces health care related financial burdens to poor households [34]

Resource allocation in many developing health systems depends on health budget distribution by central government. It is imperative that ways of examining socioeconomic disparities in health conditions and service delivery are used to examine population access to health programmes [35], and to inform policy debate and resource allocation. In Tanzania, the health budget, except for salaries, is allocated centrally on the basis of need, where the allocation formula is driven by four main components: population size, which accounts for 70% of the budget; percentage of population below the poverty line; transport needs (district vehicle route) and average under-fives mortality (used as a proxy for burden of disease), which each accounts for 10% [36]. Given the current mortality and coverage rates per quintiles, one can question whether the current allocation formula sufficiently incorporates concerns for equity. Populated and richer urban districts are likely to receive more funding from central government than rural districts. Incorporating measures of inequity such as the Gini coefficient in the resource allocation formula would explicitly address the health care needs of the worst-off [37].

In interpreting the results of this study, caution should be exercised. Our findings have affirmed that modelling tools such as LiST can be used to generate policy options to aid efficient allocation of limited health care resources. However, even if our modelling on health and equity impact is based on the most recent and best available evidence, our estimates are uncertain and can never be better than the assumptions they rest on. Moreover, we have not estimated the costs of achieving high coverage rates for the worst off quintiles. The estimate of the predicted impact on mortality relies on adherence to the standard quality of medical care. The ambitious scale up in this paper would require substantial investment in the health system and assumes that high quality services could be implemented everywhere and for everyone. This assumption may not hold true. Even if absolute effectiveness is highest in the groups with highest mortality, cost-effectiveness analysis of these interventions for these sub-groups may change the picture. An extended cost-effectiveness analysis is therefore the next logical step from our findings here.

## Conclusions

This study has given an account of how maternal and child health MGDs might be achieved by addressing the health care needs of the worst-off population. The use of scientific evidence to inform policy debates is likely to aid key policy decisions such as training and fair allocation of human resource for health, efficient health financing and expanding community based health care to reach all population. Informed policy choices affecting sub-groups of the population is central to rapid scale up of maternal and child health interventions within a framework of universal health care for all.

## Abbreviations

MDG: Millennium Development Goals; LiST: Life Saved Tool; TDHS: Tanzania Demographic and Health Survey; MMR: Maternal Mortality Ratio (per 100,000 live births); U5MR: Under five mortality rate (per 1000 live births).

## Competing interests

The authors declare that they have no competing interests.

## Authors’ contributions

GMR and OFN: designed the study, acquired the data, analysed and interpreted the results and drafted the manuscript. FNG: contributed in data analysis and manuscript writing. All authors read and approved the final manuscript.

## Pre-publication history

The pre-publication history for this paper can be accessed here:

http://www.biomedcentral.com/1471-2458/12/1119/prepub
